# Hepatitis B vaccination uptake in hard-to-reach populations in London: a cross-sectional study

**DOI:** 10.1186/s12879-019-3926-2

**Published:** 2019-05-02

**Authors:** Josephine E. B. Taylor, Julian Surey, Jennifer MacLellan, Marie Francis, Ibrahim Abubakar, Helen R. Stagg

**Affiliations:** 1 Institute for Global Health, University College London, 4th floor, Mortimer Market Centre, off Capper Street, London, WC1E 6JB UK; 2 Usher Institute of Population Health Sciences and Informatics, The University of Edinburgh, 30 West Richmond Street, Edinburgh, EH8 9DX UK

**Keywords:** Hepatitis B virus, Vaccination, Hard-to-reach, Prisoners, Homeless, Injecting drug users

## Abstract

**Background:**

In the UK, hepatitis B virus (HBV) incidence is associated with migrants from particular high-burden countries and population groups deemed ‘hard-to-reach’ by standard healthcare services: the homeless, people who inject drugs and ex-prisoners. Currently, there is a national targeted HBV vaccine policy for such at-risk groups, but there is limited recent evidence about 1) the levels of vaccine uptake, 2) the factors associated with incomplete vaccination, and 3) reasons for incomplete vaccination.

**Methods:**

A questionnaire capturing social and medical history, demographic factors and information about HBV vaccination status was completed by individuals deemed hard-to-reach due to socio-structural factors that criminalise, isolate and stigmatise who consented to participate in a randomised controlled trial of a peer intervention to promote engagement with hepatitis C services. The questionnaire also captured the reasons for incomplete vaccination. Descriptive, univariable and multivariable regression analyses were undertaken.

**Results:**

Three hundred fourty six participants completed the questionnaire. 1) 52.3% (*n* = 181) reported full HBV vaccination. 2) Within a multivariable model adjusting for sociodemographic variables, the presence of one or two or more socio-structural factors that are included in the national targeted vaccination policy was associated with protection against incomplete HBV vaccination (51.7% vaccine coverage in those with one factor, odds ratio 0.43 [95% confidence interval 0.20–0.92]); 70.1% coverage with two or more factors, 0.19 [0.09–0.39]; overall *p*-value < 0.001). Being female was also associated with lower vaccine uptake (2.37 [1.24–4.57], 0.01). Examining the socio-structural factors individually, intravenous drug use was associated with protection against incomplete HBV vaccination. 3) The most common reasons declared for incomplete vaccination were never being offered the vaccine or not returning for further doses.

**Conclusion:**

Within this study of HBV vaccination uptake among hard-to-reach population groups in London, UK, we document 52.3% coverage of the full vaccine course. Critically, although participants recommended for immunisation within national guidelines had an increased likelihood of receiving a complete vaccine course, we note surprisingly low coverage in the presence of the risk factors that are national indicators for vaccination. Public health bodies should make additional efforts to improve coverage in the hard-to-reach through improved vaccine delivery systems.

**Trial registration:**

ISRCTN24707359, Registered 19th October 2012.

**Electronic supplementary material:**

The online version of this article (10.1186/s12879-019-3926-2) contains supplementary material, which is available to authorized users.

## Background

The global burden of hepatitis B virus (HBV) is substantial, with 257 million people living with chronic infection globally [1]. Chronic infections can lead to cirrhosis and hepatocellular carcinoma and cause around 500,000–700,000 deaths a year [2]. In the UK in 2017, the incidence of infection was low at 0.80/100,000, [3] but it was concentrated in specific population groups, including migrants from high burden countries and individuals deemed ‘hard-to-reach’ by healthcare services due to socio-structural factors that criminalise, isolate and stigmatise, such as homelessness, injecting drug use (IDU) and imprisonment [4, 5]. In order to fulfil its commitment to viral hepatitis elimination by 2030, the UK needs to effectively address infection prevention and treatment in these groups [6].

A HBV subunit vaccine has been available since 1982 and is 95% effective at preventing infection in people who have completed the three dose schedule (zero, one and six months) [7]. A rapid schedule can also be used (zero, seven and 21 days), with a fourth dose recommended for ongoing protection [8]. In the UK, HBV vaccination has historically been targeted towards population groups such as people who inject drugs (PWIDs), individuals who change sexual partners frequently (particularly men who have sex with men), patients with chronic liver disease (particularly with chronic hepatitis C virus infection [HCV]), prisoners and those at occupational risk. These target groups are laid out in the Green Book, published by Public Health England (PHE) [8]. Since the autumn of 2017, HBV vaccination for all children aged one to 10 has also been incorporated into the UK immunisation schedule [8].

Although homelessness is not a specific indicator for HBV vaccination in the UK, this population group overlaps with the target groups of the Green Book. Previous research has highlighted the association between homelessness and tri-morbidity (the combination of physical and mental ill-health and substance abuse), unprotected sex, exposure to blood-borne viruses and IDU [9–14]. The cycle of homelessness and imprisonment is also well documented [15–17]. The homeless are disproportionately represented in the population of those experiencing the criminal justice system and homelessness can be a major factor in both offending and re-offending. There is often a lack of housing and support options available to individuals leaving custody. It has also been suggested that the homeless will re-offend to keep a roof over their head.

The latest figures estimate the homeless population in London to be over 164,000, with around 3% living in single bed homeless hostels [18]. The majority of the rough-sleeping population in London identify as White British and White Other (35 and 28%, respectively) [19]. The nationalities of the 46% of non UK-born rough sleepers is diverse. In recent years, the proportion of rough sleepers from Central and Eastern European Countries has declined (23% in 2017/18 compared to 37% in 2015/16). People born in Romania and Poland make up the two largest non-UK nationalities (9 and 8% respectively) [19].

Individuals within these population groups often have difficulties in accessing traditional healthcare systems [5, 20]. Outreach services such as ‘Find and Treat’ in London- which seeks to detect tuberculosis in hard-to-reach groups and support people through diagnosis and treatment- exist to meet the health needs of vulnerable individuals by engagement with homeless shelters, etc., but there is often a lack of coordination between boroughs and the National Health Service (NHS) [21].

In order to understand how effectively the national public health vaccination policy documented in the Green Book is being implemented among risk groups, this study aimed to assess the levels of HBV vaccine coverage in a hard-to-reach population in London, the epidemiological factors associated with coverage, and the individual reasons for not completing the vaccine course.

## Methods

### Study design and participants

The HALT: Hepatitis trial was a randomised non-blinded controlled trial to assess the efficacy of a Peer Advocacy intervention to promote engagement with healthcare services in individuals chronically infected with viral hepatitis [22]. Potential trial participants were approached through outreach services such as drug treatment centres, homeless hostels and day centres across London, UK between August 2013 and June 2015. The eligibility criteria were being hard-to-reach by normal healthcare services (evidenced by engagement with outreach services as a client), over the age of 16 years, and providing written informed consent. Individuals were excluded if they were already on treatment for hepatitis C virus (HCV) or HBV. In order to fully enrol in the trial, consenting participants had to test positive HBV or HCV infection.

This study is a cross-sectional analysis of the data collected at baseline from the potential trial participant population i.e. it does not exclude anyone by HBV or HCV status. A questionnaire captured self-reported demographic and clinical information, including whether participants had been vaccinated against HBV and how many doses they had received (Additional file 1). Participants were also asked to select a reason for not receiving the vaccine or receiving less than three doses.

### Study outcome

The outcome of interest was having an incomplete self-declared HBV vaccination history (less than three doses). Self-declared HBV vaccination status was classified as: no doses, one dose, two doses, three or more doses, or unknown status. For the regression model, zero to two doses were combined into a single stratum (incomplete vaccination status).

### Exposures of interest

Homelessness was collated into a variable with four strata: unknown, never homeless, ever homeless, or currently homeless. Current homelessness outranked previous homelessness. Drug use was categorised as current IDU, history of IDU, non-IDU (this captured those who stated that they had used drugs non-intravenously in the past or currently), no drug use, and unknown. Current IDU outranked history of IDU, IDU outranked non-IDU. Imprisonment was categorised into ever imprisoned, ever arrested but not imprisoned, neither, and unknown. Excessive alcohol consumption and smoking history was documented as present, absent, and unknown. Information about smoking combined into the single variable ‘smoking history’, which combined questions on the number of cigarettes a day and current or previous smoking to yield the strata Present, Absent, and Unknown.

Given the critical nature of the Green Book in providing vaccination guidance [8], the presence of zero, one, or two or more Green Book risk factors that should trigger the offer of vaccination were recorded in a single variable. The factors included were thus socio-structural factors that criminalise and stigmatise i.e. drug use, homelessness, imprisonment, being arrested, and liver disease (all from the baseline questionnaire), as well as HCV status derived from laboratory tests.

### Analysis

Descriptive analyses were undertaken of vaccine uptake, the number of doses taken and reasons for not accepting a full course of vaccination.

Logistic regression models were used to identify factors associated with the outcome of complete HBV vaccination using Stata version 14. Initially, univariable models were built that included the Green Book classification of target groups (by socio-structural factors). As our analysis looked at multiple potential factors associated with vaccination status, all exposures from the univariable analyses were included in the multivariable model, which was assessed for model stability and collinearity. For consistency, the univariable models were restricted to the same participants as were included in the multivariable regression. Likelihood Ratio tests (LRTs) were used to examine a priori defined potential effect modification between Green Book risk factors and homelessness, Green Book risk factors and alcohol abuse, homelessness and alcohol abuse, and ethnicity and birthplace, as well as to assess if model fit was improved by the inclusion of age as a linear or categorical variable. Later analyses looked at the individual associations between the factors documented in the Green Book and vaccine uptake.

### Ethics

This secondary data analysis was approved as part of the HALT: Hepatitis trial by the National Research Ethics Service (NRES) Committee London, Brent (ref: 13/LO/0077) and was registered as ISRCTN24707359 [23].

## Results

### Participant characteristics

Three hundred sixty four individuals consented for testing as part of the trial; of these 18 declared a previous HBV diagnosis and were thus excluded, leaving 346 (95.1%). Table 1 describes the baseline characteristics of the remaining 346 participants. The majority were male (264/346, 76.3%) and UK-born (263/346, 76.0%), with a median age of 43 years (range 19–69). 176/346 (50.9%) reported current or previous IDU, 289/346 current or previous homelessness (83.5%) and 221/346 (63.9%) imprisonment. 228/346 (65.9%) participants reported receiving a previous HBV test. 157 (45.4%) participants had two or more of the risk factors listed in the Green Book, with 246 (71.1%) reporting at least one.

**Table 1:** Baseline characteristics of the 346 participants, by number of doses of HBV vaccine

Variables	Overall		Incomplete vaccination status		Complete vaccination status		Unknown vaccination status	
	#	Col %	#	Row %	#	Row %	#	Row %
Overall	346	100	130	37.6	181	52.3	35	10.1
Gender								
Female	82	23.7	43	52.4	30	36.6	9	11.0
Male	264	76.3	87	33.0	151	57.2	26	9.8
Unknown	0	0.0	0	–	0	–	0	–
Age								
19–29	53	15.3	24	45.3	25	47.2	4	7.6
30–39	86	23.9	28	32.6	51	59.3	7	8.1
40–49	140	41.5	48	34.3	81	57.8	11	7.9
50–59	60	17.9	25	41.7	22	36.7	13	21.7
60–69	7	2.2	5	71.4	2	28.6	0	0.0
Unknown	0	0.0	0	–	0	–	0	–
Ethnicity								
White C/E European	82	23.7	33	40.2	37	45.1	12	14.6
White Other	160	46.8	53	33.1	89	55.6	18	11.3
Black African	25	7.2	16	64.0	9	36.0	0	0.0
Black Other	45	13.0	16	35.6	27	60.0	2	4.4
Mixed/Other	32	9.2	11	34.4	18	56.3	3	9.4
Unknown	2	0.6	1	50.0	1	50.0	0	0.0
Birthplace								
Not UK born	83	24.0	38	45.8	33	39.8	12	14.6
UK born	263	76.0	92	35.0	148	56.2	23	8.7
Unknown	0	0.0	0	–	0	–	0	–
Drug use								
No drug use	68	19.7	44	64.7	14	20.6	10	14.7
Non-IDU (History or current)	101	29.2	43	42.6	44	43.6	14	13.9
History of IDU	98	28.3	22	22.5	67	68.4	9	9.18
Current IDU	78	22.5	20	25.6	56	71.8	2	2.6
Unknown	1	0.0	1	100.0	0	–	0	–
Homelessness								
Never	51	14.7	26	51.0	21	41.2	4	7.8
History	104	30.1	29	27.9	63	60.6	12	11.5
Current	185	53.5	72	38.9	94	50.8	19	10.3
Unknown	6	1.2	3	50.0	3	50.0	0	–
Imprisonment/Arrested								
Neither	45	13.0	28	62.2	12	26.7	5	11.1
Arrested but not imprisoned	78	22.5	40	51.3	22	28.2	16	20.5
Imprisoned	221	64.2	61	27.6	146	66.1	14	6.3
Unknown	2	0.6	1	50.0	1	50.0	0	–
Excess alcohol consumption								
No	203	58.7	80	39.1	104	51.2	19	9.4
Yes	141	40.8	50	35.5	75	53.2	16	11.4
Unknown	2	0.6	0	–	2	100.0	0	–
Smoking History								
Absent	28	8.1	18	64.3	5	17.9	5	17.9
Present	318	91.9	112	35.2	176	55.4	30	9.4
Unknown	0	0	0	–	0	–	0	–
Liver Disease								
No	282	81.5	100	35.5	155	55.0	27	9.6
Yes	43	12.4	18	41.9	19	44.2	6	14.0
Unknown	21	6.1	12	57.1	7	33.3	2	9.5
HCV status								
Negative	247	71.4	105	42.5	114	46.2	28	11.3
Positive	95	27.4	24	25.3	65	68.4	6	6.3
Unknown	4	2.7	1	25.0	2	50.0	1	25.0
Green Book risk factors*								
0	77	22.3	45	58.4	17	22.1	15	19.5
1	89	25.7	36	40.5	46	51.7	7	7.9
2 or more	157	45.4	36	22.9	110	70.1	11	7.0
Unknown	23	6.6	13	56.5	8	34.8	2	8.7

Complete vaccination status was recorded as three or more doses. *C/E* Central and Eastern, *Col* column, *IDU* injecting drug use, *HCV* Hepatitis C virus

*Green Book risk factors include current or intermittent drug use (history of IDU and current IDU), being imprisoned, having liver disease and additionally being infected with HCV on top of having liver disease

### Level of HBV vaccination

The level of complete self-declared HBV vaccination in this cohort was 52.3% (Table 1). 227/346 (65.6%) participants stated that they had received at least one dose of HBV vaccine. Of these, 181/227 (79.7%) had a complete vaccination status (three doses), 15/227 (6.6%) reported receiving one dose of HBV vaccine and 26/227 (11.5%) reported receiving two doses. Participants reporting incomplete vaccination status (less than three doses) were more likely to be female (52%, 43/82), have never been homeless (51%, 26/51), to declare no drug use (65%, 44/68), and to have never been imprisoned or arrested (62%, 28/45). Examining the individuals with an unknown vaccination status revealed that they may be older, with a differing history of drug use, and imprisonment/arrest (Additional file 2).

When contributing risk factors were summarised into a single ‘Green Book’ variable, the percentage of participants with an incomplete vaccine status decreased from 58.4% (zero Green Book risk factors) to 40.5% (one) and 22.9% (two or more). When broken down into doses, the percentage of participants reporting zero and one dose of HBV vaccination was highest in those reporting zero Green Book risk factors (42.2 and 40.0% respectively). In comparison, with two and three or more doses, the percentage was highest in participants with two or more Green Book risk factors (50.0 and 60.8% respectively).

### Predictors of incomplete vaccine status

In univariable and multivariable models (Table 2), 63 individuals with missing data (28 missing information on exposures, 35 missing information on the outcome of interest) were excluded and age was included as a linear variable (*p* = 0.34).

**Table 2:** Factors associated with an incomplete HBV vaccination course (less than 3 doses) in hard-to-reach individuals

Variable	Univariable regression	Multivariable regression
	OR (95% CI)	OR (95% CI)
Gender	*p* < 0.001	*p* = 0.01
Male	(base)	(base)
Female	2.78 (1.59–4.89)	2.37 (1.24–4.57)
Age (linear)	*p* = 0.80	*p* = 0.07
19–29	(base)	(base)
per 10 year increase	1.03 (0.82–1.30)	1.30 (0.98–1.72)
Ethnicity	*p* = 0.29	*p* = 0.90
White C/E European	1.28 (0.69–2.36)	1.16 (0.58–2.34)
White Other	(base)	(base)
Black African	2.57 (1.04–6.38)	1.27 (0.37–4.33)
Black Other	0.91 (0.45–1.89)	0.77 (0.34–1.76)
Mixed/Other	1.03 (0.43–2.46)	0.83 (0.32–2.18)
Birthplace	*p* = 0.06	*p* = 0.65
UK born	(base)	(base)
Not UK born	1.7 (0.97–2.99)	1.19 (0.56–2.51)
Homelessness	*p* = 0.02	*p* = 0.31
Never	(base)	(base)
History	0.34 (0.16–0.74)	0.68 (0.28–1.67)
Current	0.62 (0.31–1.22)	1.11 (0.49–2.50)
Excessive alcohol consumption	p = 0.24	p = 0.65
No	(base)	(base)
Yes	0.74 (0.57–1.03)	0.88 (0.50–1.54)
Smoking history	*p* < 0.001	*p* = 0.02
Absent	(base)	(base)
Present	0.17 (0.06–0.46)	0.29 (0.09–0.89)
Green Book risk factors*	*p* < 0.001	*p* < 0.001
0	(base)	(base)
1	0.32 (0.16–0.66)	0.43 (0.20–0.92)
2 or more	0.13 (0.07–0.26)	0.19 (0.09–0.39)

All models present a complete case analysis where only participants with no missing data were included (*n = 283).* Likelihood ratio tests were used to calculate *p*-values. Multivariable model adjusts for all listed variables. *Green Book risk factors include current or intermittent drug use (history of IDU and current IDU), being imprisoned, having liver disease and additionally being infected with HCV on top of having liver disease. *C/E* Central and Eastern, *CI* Confidence Interval, *IDU* injecting drug use, *HCV* Hepatitis C virus, *OR* odds ratio

Within the univariable models, Green Book risk factors were initially examined within a single variable. Having two or more risk factors versus none was associated with protection against having an incomplete HBV vaccination status (odds ratio [OR] 0.13 [95% confidence interval {CI} 0.07–0.26). Having one risk factor was also associated with protection, albeit to a lesser degree (0.32 [0.16–0.66]), although the CIs between the two strata overlapped. Being female (2.78 [1.59–4.89], *p* < 0.001) was associated with higher odds of having an incomplete vaccination status. There was also an association between people who have smoked (either currently or in the past) with having a complete HBV vaccination status (5.73 [2.06–15.88], *p* < 0.001).

In a multivariable model, the association between Green Book risk factors and incomplete vaccination status was maintained (one factor 0.43 [0.20–0.92]); two or more factors, 0.19 [0.09–0.39]; overall *p*-value < 0.001).) Being female (2.37 (1.24–4.57), *p* = 0.01) also remained associated with incomplete vaccination.

There was no statistical evidence for interactions between Green Book risk factors and homelessness (LRT *p* = 0.79), Green Book risk factors and alcohol abuse (*p* = 0.24), ethnicity and birthplace (*p* = 0.84), or homelessness and alcohol abuse (*p* = 0.97).

Additional analyses were also undertaken examining the impact of the Green Book risk factors (drug use, imprisonment/arrest, history of liver disease, HCV status) as individual variables (Table 3). Intravenous drug use (history 0.17 [0.06–0.49]); current, 0.23 [0.08–0.62]; overall *p*-value 0.004) was associated with protection against incomplete HBV vaccination.

**Table 3:** Extended multivariable analyses, separating the Green Book risk factor exposure variable into four separate variables

Variable	OR (95% CI)
Gender	*p* = 0.005
Male	(base)
Female	2.72 (1.35–5.48)
Age (linear)	*p* = 0.12
19–29	(base)
per 10 year increase	1.22 (0.91–1.64)
Ethnicity	*p* = 0.89
White C/E European	1.12 (0.53–2.37)
White Other	(base)
Black African	1.32 (0.36–4.86)
Black Other	0.74 (0.31–1.78)
Mixed/Other	0.82 (0.30–2.28)
Birthplace	*p* = 0.66
UK born	(base)
Not UK born	0.83 (0.38–1.81)
Homelessness	*p* = 0.62
Never	(base)
History	0.72 (0.29–1.81)
Current	1.06 (0.45–2.50)
Excessive alcohol consumption	*p* = 0.53
No	(base)
Yes	0.72 (0.38–1.31)
History of smoking	*p* = 0.17
No	(base)
Yes	0.44 (0.13–1.45)
Drug Use	*p* = 0.004
No drug use	(base)
Non IDU (History or current)	0.55 (0.23–1.32)
History of IDU	0.17 (0.06–0.49)
Current IDU	0.23 (0.08–0.62)
Imprisonment/Arrested	*p* = 0.03
Neither	1.79 (0.68–4.69)
Arrested but not imprisoned	2.59 (1.27–5.29)
Imprisoned	(base)
History of liver disease	*p* = 0.14
No	(base)
Yes	1.97 (0.81–4.79)
HCV status	*p* = 0.54
Negative	(base)
Positive	1.47 (0.67–3.24)

Model presents a complete case analysis where only participants with no missing data were included (*n = 283).* Likelihood ratio tests were used to calculate p-values. *Green Book risk factors include current or intermittent drug use (history of IDU and current IDU), being imprisoned, having liver disease and additionally being infected with HCV on top of having liver disease. C/E – Central and Eastern; *CI* confidence interval, *HCV* Hepatitis C virus, *IDU* injecting drug use, *OR* odds ratio; p = *p*-value

### Reasons for incomplete vaccination

Specific reasons for not receiving any doses of HBV vaccine were declared by 85 participants (Table 4). The most common reason was never being offered vaccination, with 80% (68/85) of participants stating this as a reason for no vaccination. 41% (28/68) of participants who reported never having been offered the vaccine had at least one of the factors listed in the Green Book, identifying them as targets for the pre-exposure immunisation programme.

**Table 4:** Questionnaires captured reasons for participants not being vaccinated against HBV or not completing the schedule

Reason for no/incomplete vaccination	Frequency	Percentage (%)
No vaccination	85	100
Never been offered the vaccine	68	80
Already had Hepatitis B	8	9.4
Do not have enough time	3	3.5
Refused vaccination	2	2.4
Allergic to other vaccines/potentially this vaccine	1	1.2
Dislike needles	1	1.2
Do not believe in vaccination	1	1.2
Worried about side-effects	1	1.2
Incomplete vaccination	22	100
Did not return	13	59.1
Do not have enough time	5	22.7
Infected with Hepatitis B virus between doses	2	9.1
Side effects/allergy to first dose(s)	1	4.6
Awaiting third dose	1	4.6

Twenty two of 42 participants who had an incomplete vaccination status (one or two doses) gave a reason for not completing the vaccine schedule. The most common reason was that they did not return after their initial dose(s) (Table 4).

## Discussion

In a population of hard-to-reach individuals from homeless and outreach centres across London, the study found that only 52% had received a full three-dose course of HBV vaccine, with 74% of participants reporting at least one dose. Critically, although the proportion of individuals with an incomplete vaccine course decreased as the number of Green Book risk factors increased, two fifths of individuals lacked full vaccine protection among individuals with one factor and nearly a quarter among individuals with two or more. The presence of only a single factor should be sufficient to trigger implementation of the Green Book guidelines. Regression analyses confirmed these findings and also highlighted the association of IDU with vaccine coverage. Additionally, we found that females were more likely to have an incomplete vaccination status. The main reasons for given for participants having an incomplete vaccination status was that they had not been offered vaccination, or they had not returned to complete the schedule.

As well as detailed documentation of vaccine coverage in this critical population group for viral elimination, this is the first study, to our knowledge, to examine the relationship between Green Book risk factors and self-declared vaccine uptake. The level of uptake documented within PWIDs in our study (31%) falls within the range reported from previous studies in PWIDs (27–66%), [24–26] although it is lower than the latest figures charted by PHE in PWIDs in 2016 of 71% [27]. UK prisons have reported varying levels of HBV vaccine uptake (22–70.2%), depending on the type and location [28–31]. There is a lack of evidence of HBV vaccine uptake in the homeless, although a recent cross-sectional survey of a London homeless population has estimated the prevalence of past HBV infection as 10.4% and identified the importance of maximising vaccination uptake in this group [9]. Similarly, our study included a number of homeless people without the presence of other risk factors, who are not targets for the pre-exposure HBV immunisation programme per se.

Interestingly, we also see 22.1% of people reporting a complete vaccination status in the presence of no Green Book risk factors, potentially reflecting previous travel history, and also the nature of the population eligible for the overarching trial i.e. individuals thought to be at higher risk of chronic hepatitis C.

In agreement with previous studies in prisons, this study found females were less likely to have reported receiving three doses of HBV vaccine [29, 32]. This could be explained by both systems-related and personal factors, including healthcare workers prioritising males, as most UK hepatitis cases (70.4%) are reported in men [3].

Our study also documents the self-declared reasons of the participants about their lack of, or incomplete, HBV vaccine uptake. Critically, among the non-vaccinated, 80% declared that they had not been offered the vaccine, suggesting a lack of vaccine availability or staff knowledge of the participants and their risk factors. Similar healthcare system factors have been noted by nurses in a previous study [24].

The principle limitation is that the study relies on self-declared vaccine uptake. Due to unavailable data, sexual preferences (such as men who have sex with men and condom-less sex) could not be tested for association with HBV vaccine uptake. This study recruited a specific population that was in contact with outreach services to a trial of Peer Advocates to improve engagement with healthcare services and thus will not be representative of the entire UK hard-to-reach population. Our study undertook a complete case analysis, thus excluding data from 63 patients, of whom 35 had an unknown vaccination status. We note that the population with an unknown status may be older, with a differing history of drug use, and imprisonment/arrest, thus slightly limiting the generalisability of our findings.

Encouragingly, our analyses demonstrate that having a Green Book risk factor is associated with receipt of a full course of HBV vaccination, however, two fifths of individuals with only one Green Book risk factor reported an incomplete vaccine course and one fifth with two or more factors. More therefore needs to be done to improve coverage, particularly among groups with the risk factors that do not already lead to greater engagement with care e.g. individuals with chronic HCV or liver disease. This study has highlighted that females have an increased likelihood of incomplete vaccination, which is of particular note, given that women tend to engage better with healthcare in the general population. There was also a lack of convincing evidence of the impact HCV status or liver disease are associated with vaccination status. Together with our analysis, of the self-declared reasons for an incomplete vaccination status, this suggests that there is a need for strengthening awareness about vaccine eligibility among the relevant population groups and healthcare workers. Further qualitative research should be done to look into the barriers and enablers for returning and to help improve policy and practice. One suggestion would be incentives to help with adherence to the multi-dose vaccine schedule [33].

## Conclusions

Within this study of HBV vaccination uptake among hard-to-reach population groups in London, UK, we document approximately 50% coverage of the full vaccine course and- critically- surprisingly low coverage in the presence of the risk factors that are national indicators for vaccination. We note particular population groups associated with poorer vaccine uptake and the need for better engagement of these groups with vaccine delivery systems.

## Additional files

Additional file 1: Study Questionnaire. The study questionnaire captured social and medical history, demographic factors, information about HBV vaccination status and reasons for incomplete vaccination if applicable. It was completed by individuals consenting to participate in a randomised controlled trial of a peer intervention to promote engagement with hepatitis C services. (DOCX 824 KB)

Additional file 2: Baseline characteristics of the 346 participants, with column percentages. (DOCX 30 KB)

## Abbreviations

C/E: Central and Eastern
CI: Confidence interval
HALT: Hepatitis and Latent Tuberculosis
HBsAg: Hepatitis B surface antigen
HBV: Hepatitis B virus
HCV: Hepatitis C virus
IDU: Injecting/intravenous drug use
LR: Likelihood ratio
OR: Odds Ratio
PWID: People who inject drugs
UK: United Kingdom
