# Supplementary figures and images for: Hepatitis B vaccination uptake in hard-to-reach populations in London: a cross-sectional study
Source: BMC Infect Dis. 2019 May 2;19:372. doi: 10.1186/s12879-019-3926-2 (PMC6498651; doi:10.1186/s12879-019-3926-2)

## **Additional file 1 –** Study Questionnaire


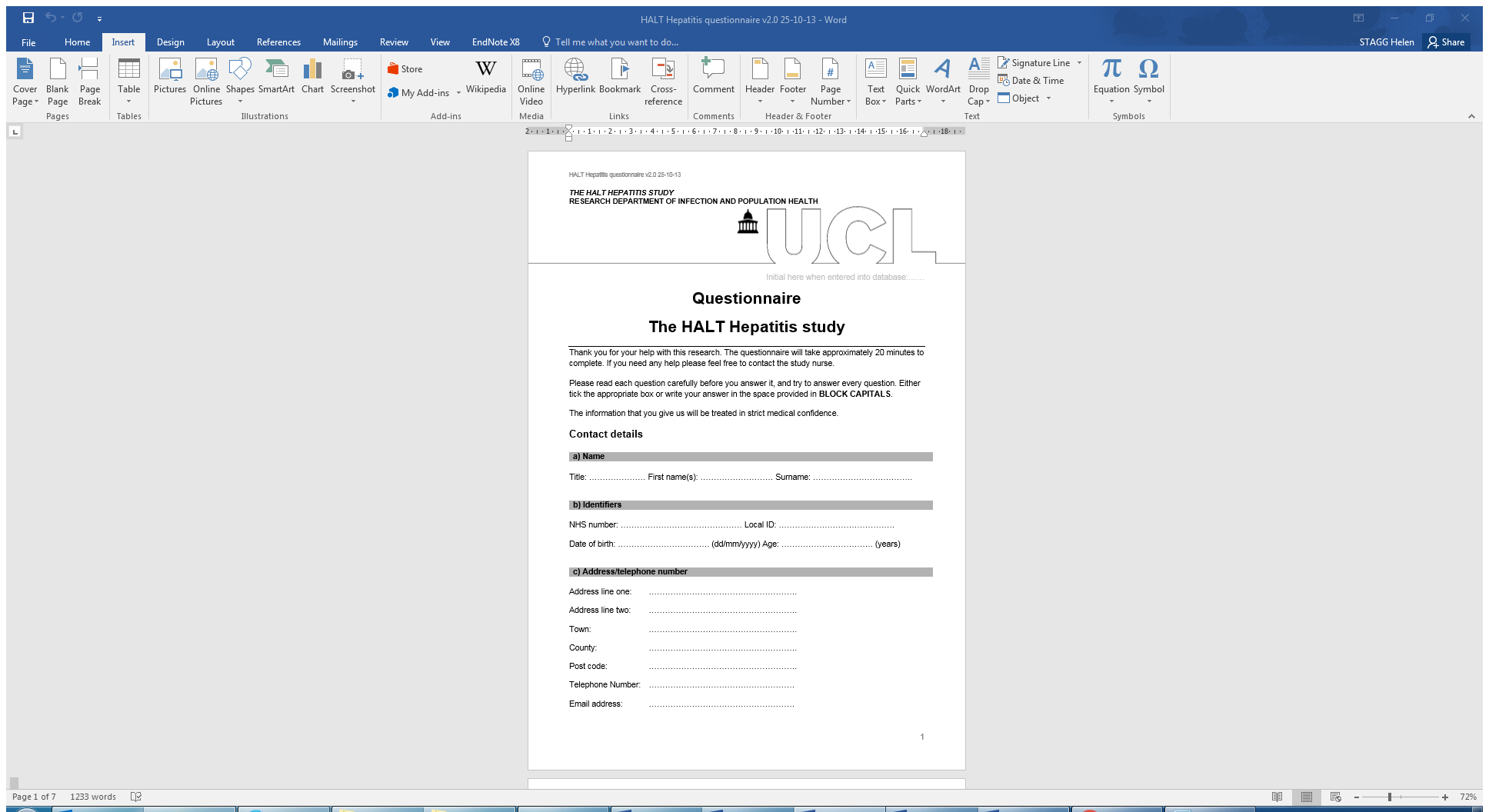


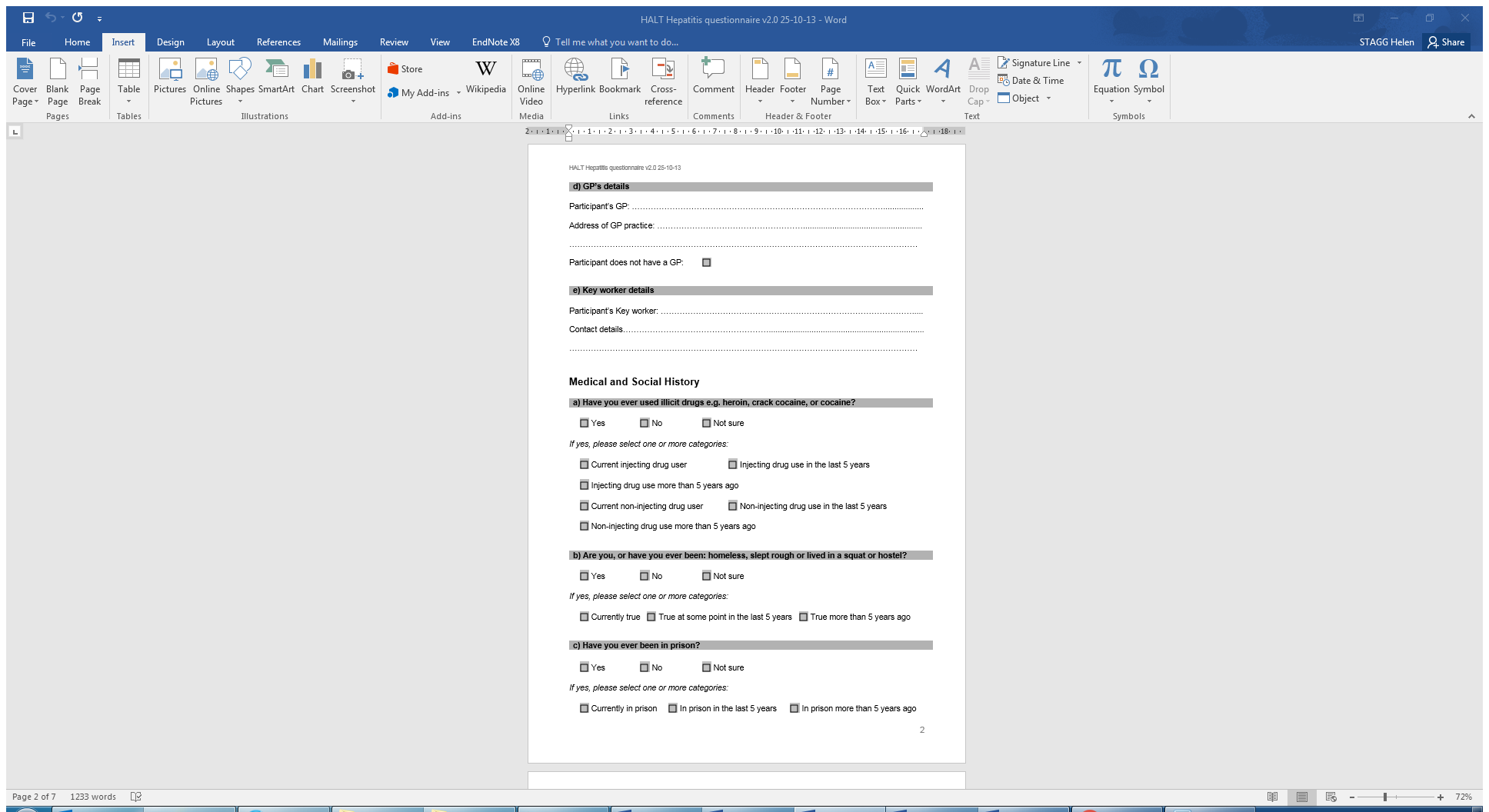


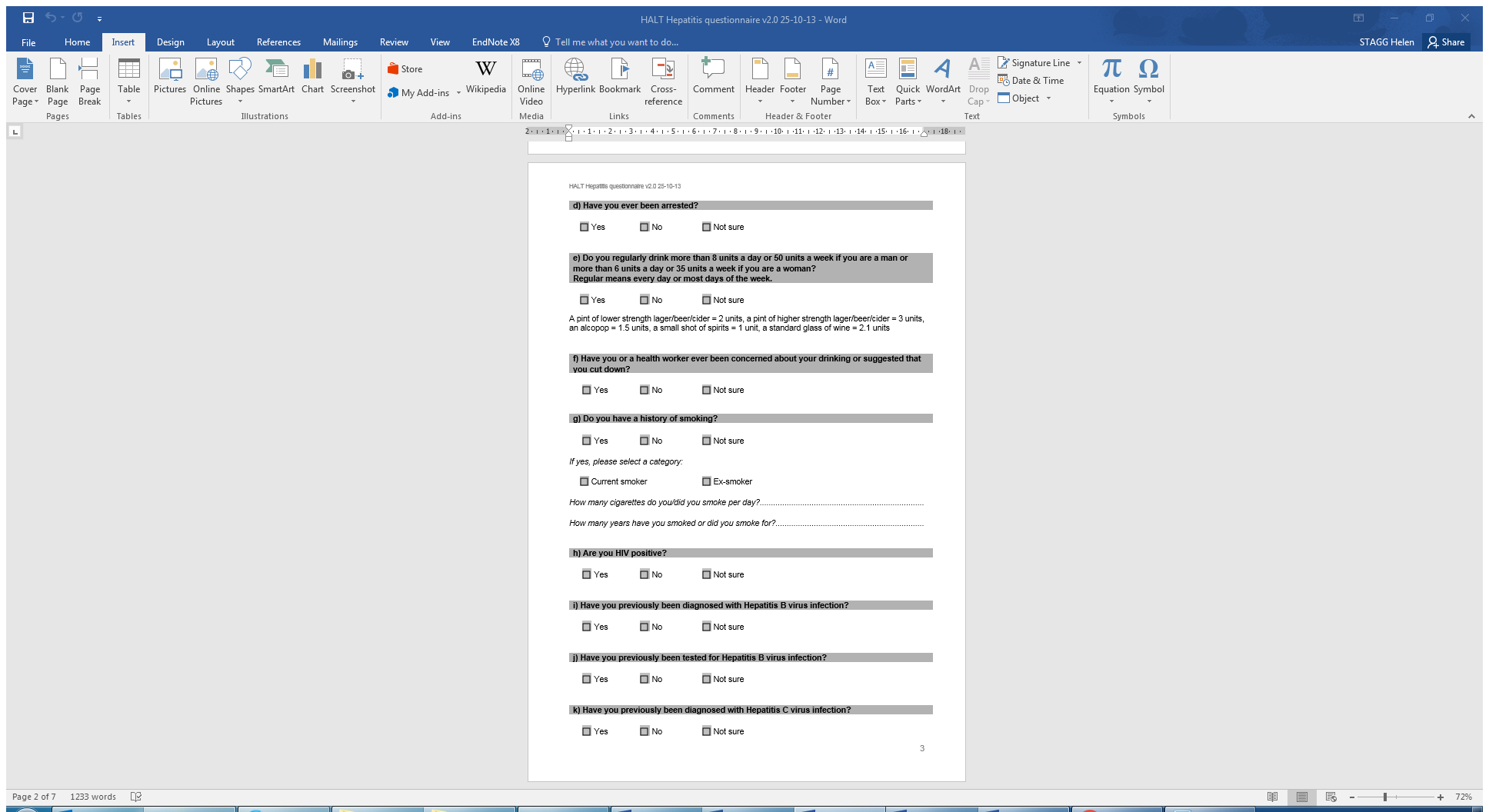


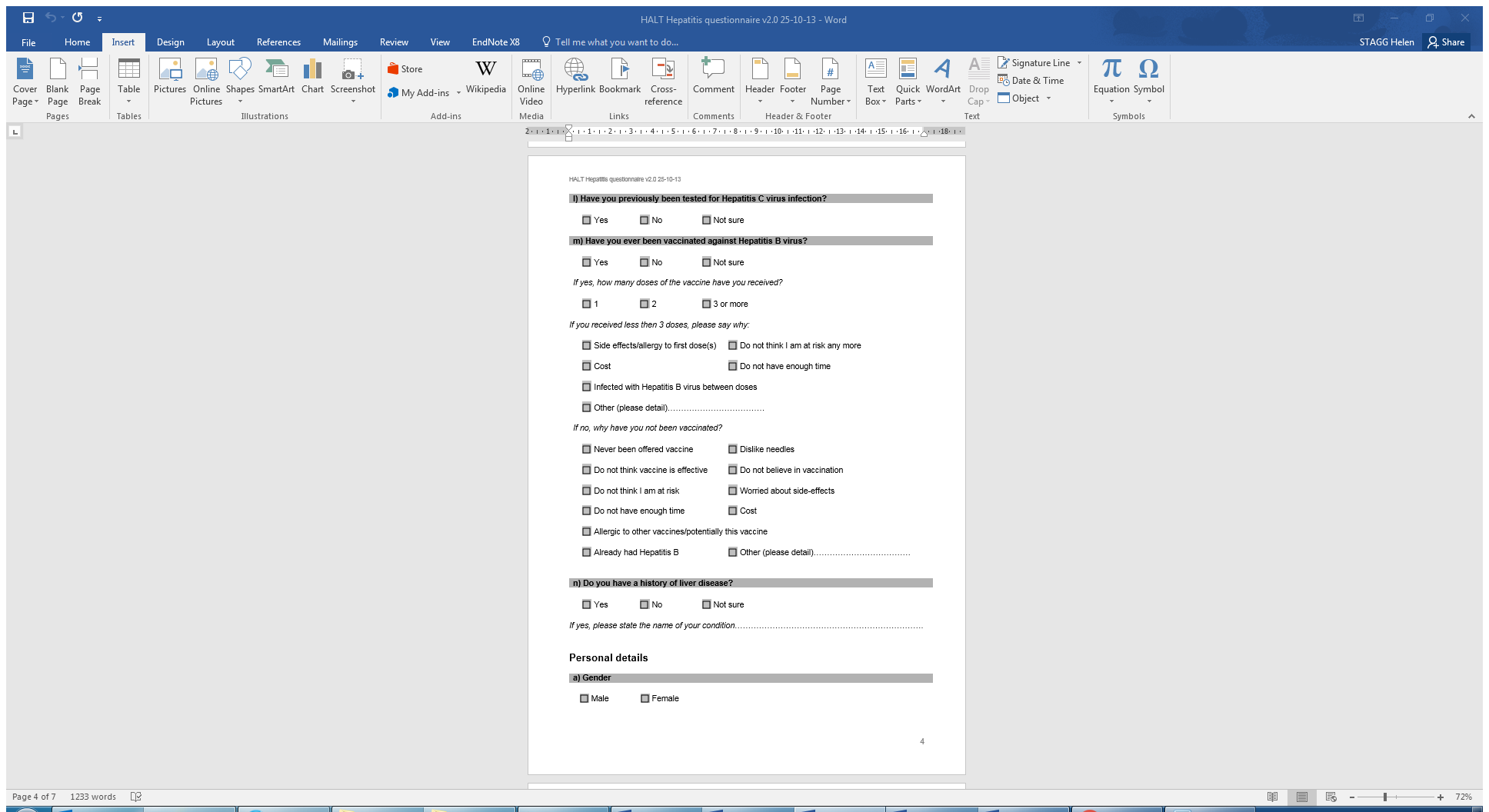


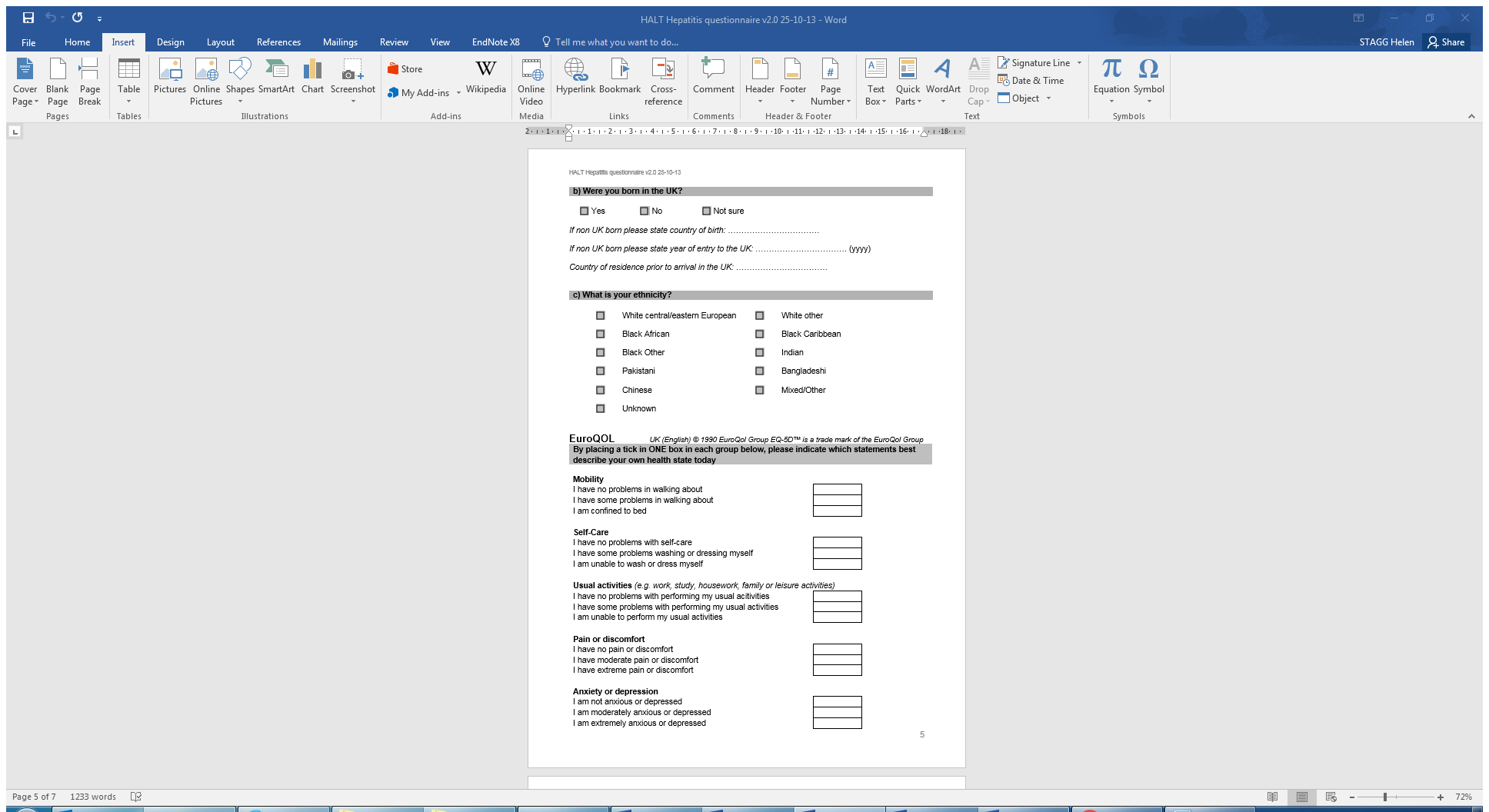


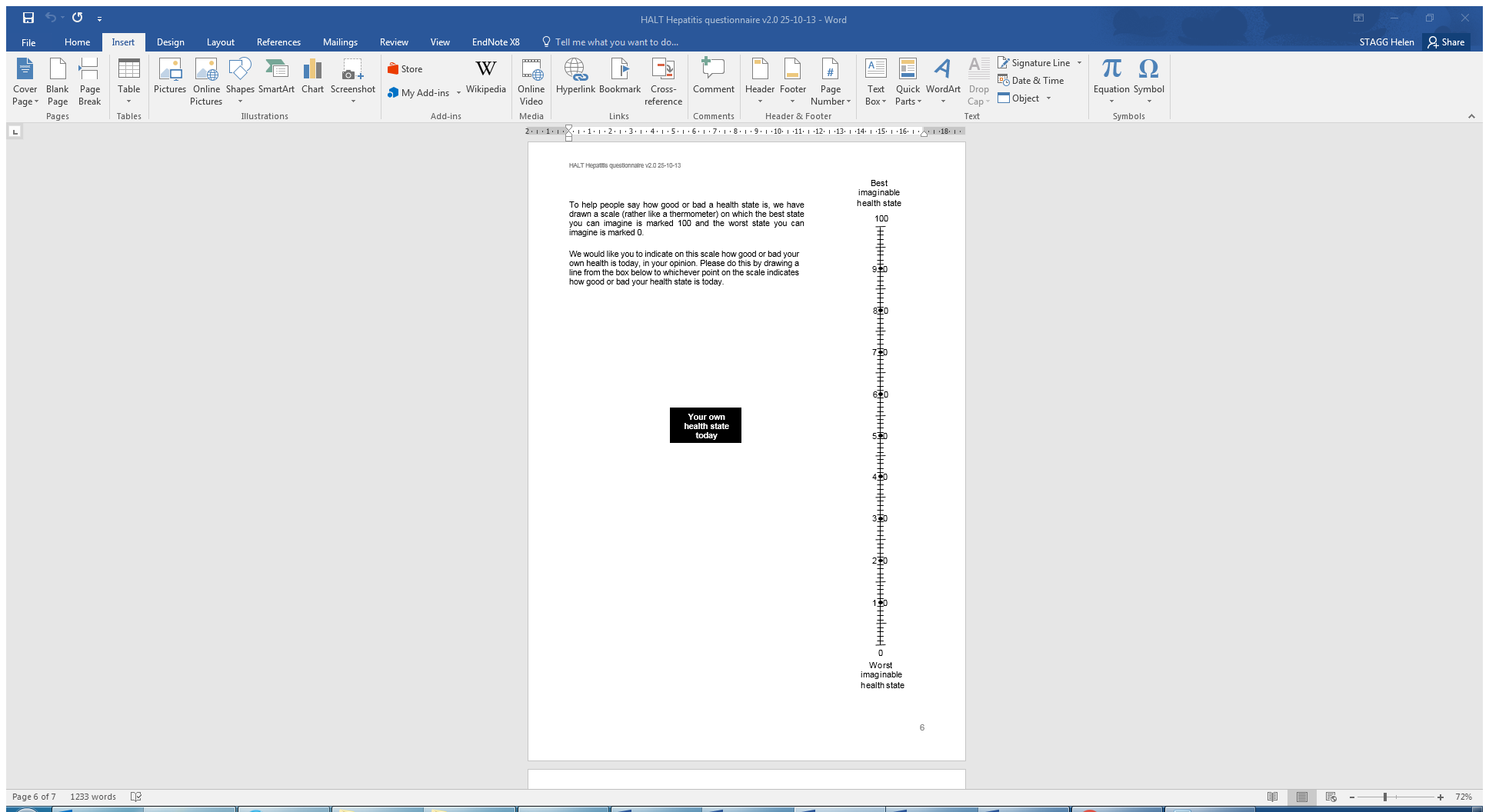


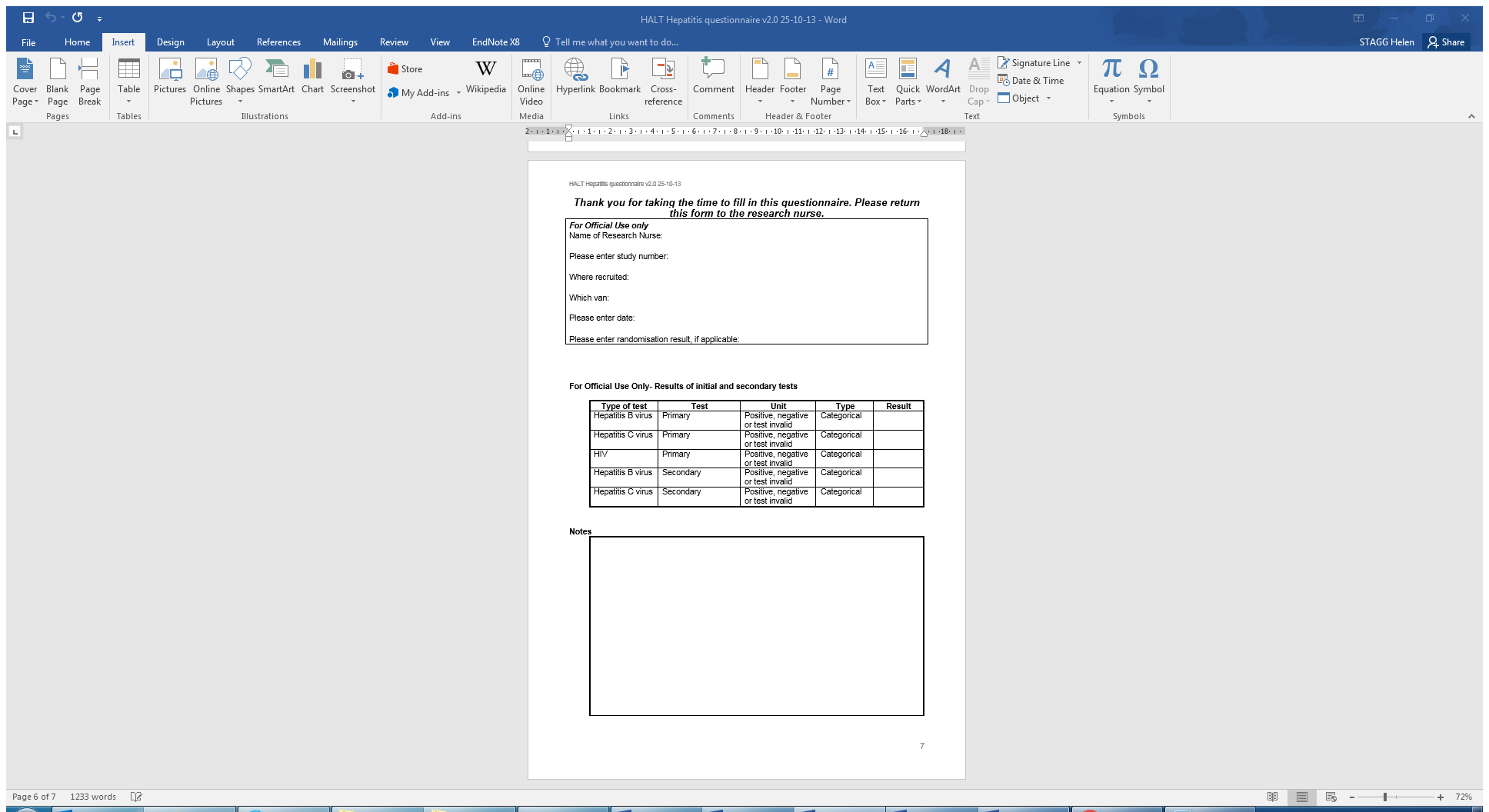

Supplement: Supplementary file 1 — Study Questionnaire. The study questionnaire captured social and medical history, demographic factors, information about HBV vaccination status and reasons for incomplete vaccination if applicable. It was completed by individuals consenting to participate in a randomised controlled trial of a peer intervention to promote engagement with hepatitis C services. (DOCX 824 KB) [file 12879_2019_3926_MOESM1_ESM.docx]
