# Supplementary material for: Hepatitis B vaccination uptake in hard-to-reach populations in London: a cross-sectional study
Source: BMC Infect Dis. 2019 May 2;19:372. doi: 10.1186/s12879-019-3926-2 (PMC6498651; doi:10.1186/s12879-019-3926-2)
Supplement: Supplementary file 2 — Baseline characteristics of the 346 participants, with column percentages. (DOCX 30 KB) [file 12879_2019_3926_MOESM2_ESM.docx]

## **Additional file 2 –** Baseline characteristics of the 346 study participants; column percentages

| **Variables** | **Overall** | | **Incomplete vaccination status** | | **Complete vaccination status** | | **Unknown vaccination status** | |
| --- | --- | --- | --- | --- | --- | --- | --- | --- |
|  | **#** | **Col %** | **#** | **Col %** | **#** | **Col %** | **#** | **Col %** |
| **Overall** | 346 | 100 | 130 | 100 | 181 | 100 | 35 | 100 |
| **Gender** |  |  |  |  |  |  |  |  |
| Female | 82 | 23.7 | 43 | 33.1 | 30 | 16.6 | 9 | 25.7 |
| Male | 264 | 76.3 | 87 | 66.9 | 151 | 83.4 | 26 | 74.3 |
| Unknown | 0 | 0.0 | 0 | 0.0 | 0 | 0.0 | 0 | 0.0 |
| **Age** |  |  |  |  |  |  |  |  |
| 19-29 | 53 | 15.3 | 24 | 18.5 | 25 | 13.8 | 4 | 11.4 |
| 30-39 | 86 | 23.9 | 28 | 21.5 | 51 | 28.2 | 7 | 20.0 |
| 40-49 | 140 | 41.5 | 48 | 36.9 | 81 | 44.8 | 11 | 31.4 |
| 50-59 | 60 | 17.9 | 25 | 19.2 | 22 | 12.2 | 13 | 37.1 |
| 60-69 | 7 | 2.2 | 5 | 3.8 | 2 | 1.1 | 0 | 0.0 |
| Unknown | 0 | 0.0 | 0 | 0.0 | 0 | 0.0 | 0 | 0.0 |
| **Ethnicity** |  |  |  |  |  |  |  |  |
| White C/E European | 82 | 23.7 | 33 | 25.4 | 37 | 20.4 | 12 | 34.3 |
| White Other | 160 | 46.8 | 53 | 40.8 | 89 | 49.2 | 18 | 51.4 |
| Black African | 25 | 7.2 | 16 | 12.3 | 9 | 5.0 | 0 | 0.0 |
| Black Other | 45 | 13.0 | 16 | 12.3 | 27 | 14.9 | 2 | 5.7 |
| Mixed/Other | 32 | 9.2 | 11 | 8.5 | 18 | 9.9 | 3 | 8.6 |
| Unknown | 2 | 0.6 | 1 | 0.8 | 1 | 0.6 | 0 | 0.0 |
| **Birthplace** |  |  |  |  |  |  |  |  |
| Not UK born | 83 | 24.0 | 38 | 29.2 | 33 | 18.2 | 12 | 34.3 |
| UK born | 263 | 76.0 | 92 | 70.8 | 148 | 81.8 | 23 | 65.7 |
| Unknown | 0 | 0.0 | 0 | 0.0 | 0 | 0.0 | 0 | 0.0 |
| **Drug use** |  |  |  |  |  |  |  |  |
| No drug use | 68 | 19.7 | 44 | 33.8 | 14 | 7.7 | 10 | 28.6 |
| Non-IDU (History or current) | 101 | 29.2 | 43 | 33.1 | 44 | 24.3 | 14 | 40.0 |
| History of IDU | 98 | 28.3 | 22 | 16.9 | 67 | 37.0 | 9 | 25.7 |
| Current IDU | 78 | 22.5 | 20 | 15.4 | 56 | 30.9 | 2 | 5.7 |
| Unknown | 1 | 0.0 | 1 | 0.8 | 0 | 0.0 | 0 | 0.0 |
| **Homelessness** |  |  |  |  |  |  |  |  |
| Never | 51 | 14.7 | 26 | 20.0 | 21 | 11.6 | 4 | 11.4 |
| History | 104 | 30.1 | 29 | 22.3 | 63 | 34.8 | 12 | 34.3 |
| Current | 185 | 53.5 | 72 | 55.4 | 94 | 51.9 | 19 | 54.3 |
| Unknown | 6 | 1.2 | 3 | 2.3 | 3 | 1.7 | 0 | 0.0 |
| **Imprisonment/Arrested** |  |  |  |  |  |  |  |  |
| Neither | 45 | 13.0 | 28 | 21.5 | 12 | 6.6 | 5 | 14.3 |
| Arrested but not imprisoned | 78 | 22.5 | 40 | 30.8 | 22 | 12.2 | 16 | 45.7 |
| Imprisoned | 221 | 64.2 | 61 | 46.9 | 146 | 80.7 | 14 | 40.0 |
| Unknown | 2 | 0.6 | 1 | 0.8 | 1 | 0.6 | 0 | 0.0 |
| **Excess alcohol consumption** |  |  |  |  |  |  |  |  |
| No | 203 | 58.7 | 80 | 61.5 | 104 | 57.5 | 19 | 54.3 |
| Yes | 141 | 40.8 | 50 | 38.5 | 75 | 41.4 | 16 | 45.7 |
| Unknown | 2 | 0.6 | 0 | 0.0 | 2 | 1.1 | 0 | 0.0 |

**Table 5, continued**

| **Variables** | **Overall** | | **Incomplete vaccination status** | | **Complete vaccination status** | | **Unknown vaccination status** | |
| --- | --- | --- | --- | --- | --- | --- | --- | --- |
|  | **#** | **Col %** | **#** | **Col %** | **#** | **Col %** | **#** | **Col**  **%** |
| **Overall** | 346 | 100 | 130 | 100 | 181 | 100 | 35 | 100 |
|  |  |  |  |  |  |  |  |  |
| **Smoking History** | 28 | 8.1 | 18 | 64.3 | 5 | 17.9 | 5 | 17.9 |
| Absent | 28 | 8.1 | 18 | 13.8 | 5 | 2.8 | 5 | 14.3 |
| Present | 318 | 91.9 | 112 | 86.2 | 176 | 97.2 | 30 | 85.7 |
| Unknown | 0 | 0.0 | 0 | 0.0 | 0 | 0.0 | 0 | 0.0 |
| **Liver Disease** |  |  |  |  |  |  |  |  |
| No | 282 | 81.5 | 100 | 76.9 | 155 | 85.6 | 27 | 77.1 |
| Yes | 43 | 12.4 | 18 | 13.8 | 19 | 10.5 | 6 | 17.1 |
| Unknown | 21 | 6.1 | 12 | 9.2 | 7 | 3.9 | 2 | 5.7 |
| **HCV status** |  |  |  |  |  |  |  |  |
| Negative | 247 | 71.4 | 105 | 80.8 | 114 | 63.0 | 28 | 80.0 |
| Positive | 95 | 27.5 | 24 | 18.5 | 65 | 35.9 | 6 | 17.1 |
| Unknown | 4 | 1.2 | 1 | 0.8 | 2 | 1.1 | 1 | 2.9 |
| **Green Book risk factors*** |  |  |  |  |  |  |  |  |
| 0 | 77 | 22.3 | 45 | 34.6 | 17 | 9.4 | 15 | 42.9 |
| 1 | 89 | 25.7 | 36 | 27.7 | 46 | 25.4 | 7 | 20.0 |
| 2 or more | 157 | 45.4 | 36 | 27.7 | 110 | 60.8 | 11 | 31.4 |
|  | | | | | | | | |

Complete vaccination status was recorded as three or more doses. C/E – Central and Eastern; Col.- column; IDU – injecting drug use; HCV – Hepatitis C virus

*Green Book risk factors include current or intermittent drug use (history of IDU and current IDU), being imprisoned, having liver disease and additionally being infected with HCV on top of having liver disease.
